# Comparison of remimazolam and midazolam for sedation during colonoscopy in Japanese patients: A propensity score matching analysis

**DOI:** 10.1002/deo2.412

**Published:** 2024-07-23

**Authors:** Kanako Ogura, Ryoji Ichijima, Hisatomo Ikehara, Tomomi Sugita, Daisuke Yamaguchi, Yasuhiko Nagata, Mitsuru Esaki, Yosuke Minoda, Hiroyuki Ono, Kinichi Hotta, Shinsuke Kiriyama, Tetsuya Sumiyoshi, Yuichi Kanmura

**Affiliations:** ^1^ Department of Medicine Nihon University School of Medicine Division of Gastroenterology and Hepatology Tokyo Japan; ^2^ Department of Gastroenterology Nagata Surgery and Gastroenterological Clinic Tokyo Japan; ^3^ Department of Gastroenterology Kiriyama Clinic Gunma Japan; ^4^ Department of Gastroenterology Internal Medicine Kitasato University School of Medicine Kanagawa Japan; ^5^ Department of Gastroenterology National Hospital Organization Ureshino Medical Center Saga Japan; ^6^ Department of Medicine and Bioregulatory Science Graduate School of Medical Sciences Kyushu University Fukuoka Japan; ^7^ Division of Endoscopy Shizuoka Cancer Center Shizuoka Japan; ^8^ Department of Gastroenterology Tonan Hospital Hokkaido Japan; ^9^ Department of Anesthesiology Fujimoto General Hospital Miyazaki Japan

**Keywords:** colonoscopy, midazolam, propensity score, remimazolam, sedation

## Abstract

**Objectives:**

To compare the efficacy and safety of sedation with midazolam and remimazolam for colorectal endoscopy.

**Methods:**

This single‐center, two‐arm, post‐hoc analysis of the REM‐IICTJP01 study investigated the efficacy and safety of remimazolam for gastrointestinal endoscopic sedation. We enrolled 40 and 208 patients who underwent colonoscopy under remimazolam and midazolam sedation, respectively, during the same period. The primary outcome was the time from the end of the colonoscopy until discharge. The secondary outcomes included the time from the end of the colonoscopy until awakening, dosage, and adverse events. Propensity score matching was employed to eliminate the effect of confounding factors.

**Results:**

Thirty‐seven patients in each group were matched. After propensity matching, the time to awakening after colonoscopy was 28.0 (13.0–37.0) min in the midazolam group and 0 (0–0) min in the remimazolam group; moreover, the time till discharge was 40.0 (35.0–46.5) min in the midazolam group and 0 (0–5.0) min in the remimazolam group, both of which were significantly shorter in the remimazolam group (*p* < 0.01). The number of additional doses was 0 (0–0) and 2 (1–3) in the midazolam and remimazolam groups, respectively. The total dose was 2.0 (2.0–3.5) and 6.0 (5.0–7.0) mg in the midazolam and remimazolam groups, respectively.

**Conclusions:**

Remimazolam yielded significantly faster times to awakening and discharge safely compared to midazolam.

## INTRODUCTION

The incidence of colorectal cancer has increased in recent years, and colonoscopy is recommended for early detection and treatment.^1^, ^2^, ^3^ A colonoscopy is an invasive examination for the patient and can be painful depending on their physique and history of previous abdominal surgery. Thus, there is a growing demand for colonoscopy under sedation to improve tolerability.^4^ However, unlike Western countries, the clinical environment related to endoscopic sedation is still in the nascent stage in Japan. Endoscopists themselves administer sedation due to a shortage of anesthesiologists. Moreover, the Japanese National Health Insurance covers only a few sedatives for gastrointestinal endoscopy, leading to the off‐label use of benzodiazepines. Currently, midazolam is the recommended benzodiazepine, which is frequently used in routine clinical practice.^4^ However, midazolam has a long half‐life, and its sedative effect is sustained, even after completing the examination procedure, prolonging the time to discharge.^5^, ^6^ This increases the requirement for recovery rooms; thus, the use of sedatives must be limited owing to the limited availability of recovery beds. This situation is further complicated by the many incidents of patients suffering falls after leaving the colonoscopy room owing to the policy encouraging early release.

Remimazolam is a newly developed ultra‐short‐acting benzodiazepine. It has been approved by the US Food and Drug Administration and is used for sedation during gastrointestinal endoscopy; however, it is not yet covered by Japan's insurance. Remimazolam has a shorter pharmacokinetic half‐life than midazolam, which is expected to shorten the time to awakening after the end of the examination and to discharge from the recovery room; moreover, it may be useful in overcoming the hurdles related to sedation in the field of endoscopy.^7^, ^8^, ^9^, ^10^, ^11^, ^12^, ^13^, ^14^, ^15^

We have previously reported on the utility of remimazolam in the REM‐IICT JP01 study, an investigator‐initiated clinical trial examining endoscopic sedation using remimazolam.^16^, ^17^ Although previous studies have reported the safety and utility of remimazolam,^9^, ^12^, ^18^, ^19^, ^20^ they did not compare remimazolam with other drugs, and currently, no research has compared remimazolam with midazolam in colonoscopies performed in Japan. Therefore, this study entailed a subanalysis of the REM‐IICTJP01 study and compared the efficacy and safety of midazolam and remimazolam in colorectal endoscopy.

## METHODS

### Study design

The REM‐IICT JP01 study was registered with the Japan Registry of Clinical Trials (trial number: jRCT2031200360) on February 15, 2021. This study was a single‐center, two‐arm, post‐hoc analysis of the REM‐IICT‐JP01 study and was approved by the institutional review board of Nihon University Hospital. The study was conducted in accordance with the Declaration of Helsinki. Informed written consent was obtained from all participants before the start of endoscopy.

### Patients

**FIGURE 1:**
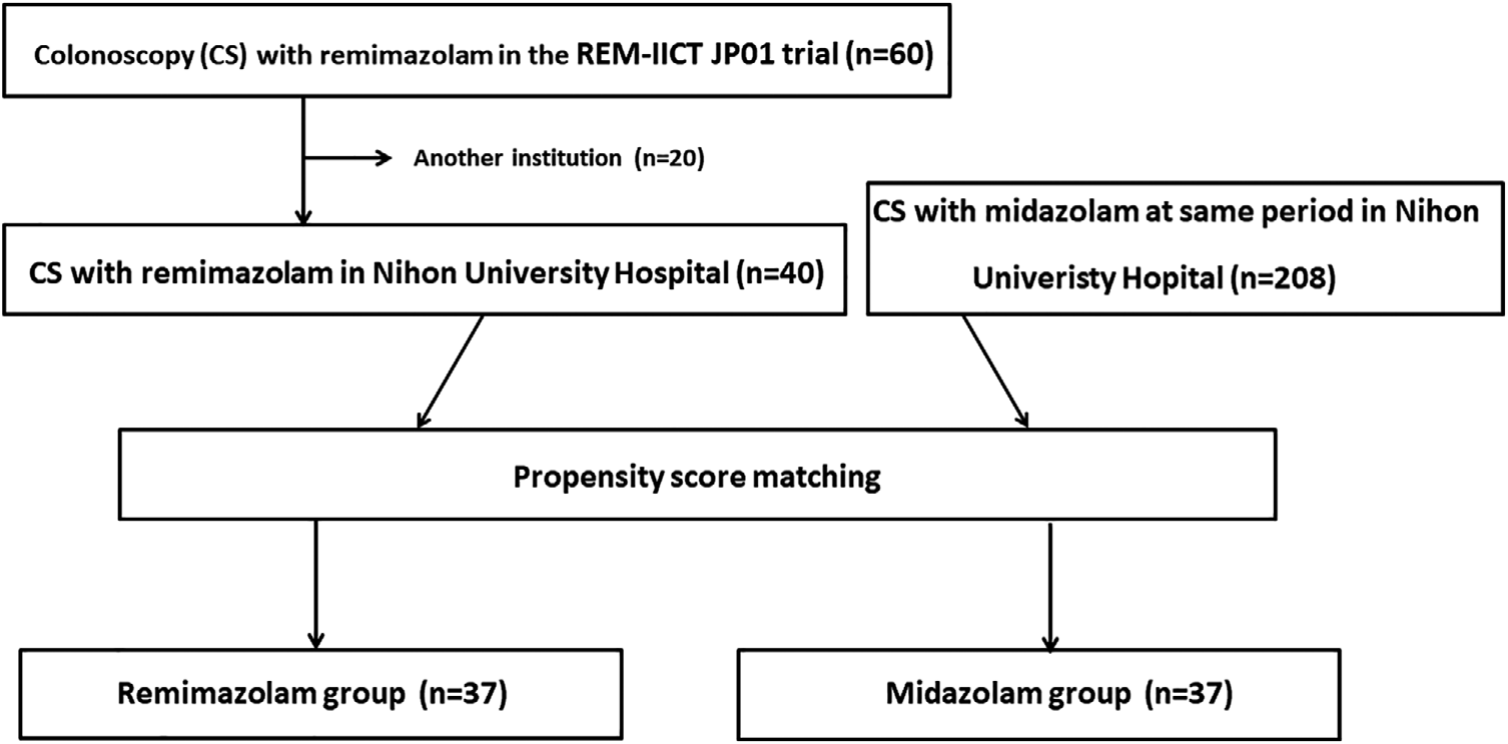
Flow chart of the study selection process. CS, colonoscopy; EGD, esophagogastroduodenoscopy; BMI, body mass index; and ASA, American Society of Anesthesiologists.

Forty patients from the remimazolam group, part of the REM‐IICT‐JP01 study on remimazolam's efficacy and safety for gastrointestinal endoscopic sedation, underwent colonoscopy under remimazolam sedation at the Nihon University Hospital between April 1, 2021, and December 31, 2021. Concurrently, 208 patients who received midazolam sedation for their colonoscopies at the same hospital were designated as the midazolam group (Figure 1).

### Inclusion and exclusion criteria

Patients were allocated to the remimazolam group if they met the criteria established in the protocol of the REM‐IICT‐JP01 trial.^16^, ^17^ The eligibility criterion was Japanese patients with a body mass index (BMI) <30 kg/m^2^ scheduled to undergo a colonoscopy without concomitant analgesia. The main exclusion criteria were: (1) regular use of benzodiazepines; (2) patients on analgesics; (3) patients with severe psychiatric disorders; (4) history of abdominal surgery; (5) patients with severe respiratory disease; (6) patients with a SpO_2_ <95% (room air) at the time of screening and during the study drug administration period (prior to the start of study drug administration); (7) patients with a Mallampati classification of III or higher; (8) history of sleep apnea; (9) patients with any of the following clinical laboratory parameters during the screening period: aspartate transaminase levels ≥2.5 times the upper limit of the institutional reference value, alanine transaminase levels ≥2.5 times the upper limit of the institutional reference value, total bilirubin levels ≥1.5 times the upper limit of the institutional reference value; (10) women who were pregnant, lactating, or who may be pregnant. The midazolam group included all patients who underwent colonoscopy under sedation with midazolam without analgesics at our hospital during the same period.

### Endoscopic procedure and drug administration

#### Before endoscopy

Patients in the remimazolam and midazolam groups abstained from eating from 9 p.m. on the day before colonoscopy. A 20‐mL solution of picosulfate sodium hydrate was administered as a pretreatment at 8:00 p.m. one day before the examination, and a bowel lavage solution (2–4 L of Nifrec or 1.5–2 L of Mobiprep) was administered 4 h before the initiation of examination on the day of colonoscopy. An antispasmodic (butylscopolamine bromide 10–20 mg or glucagon 0.5–1 mg) was administered intravenously at the start of the examination, if necessary.

Remimazolam or midazolam was administered intravenously as a single initial dose. The dose of remimazolam was determined according to the REM‐IICT‐JP01 protocol, while an initial dose of midazolam of 2–3 mg was administered at the endoscopist's discretion. The sedation level was assessed using the Modified Observer's Assessment of Alertness and Sedation (MOAA/S) Scale^21^ at an interval of at least 2 min from the start of the first dose, and sedation (MOAA/S score ≤4) was confirmed before the initiation of colonoscopy. If sedation was not achieved, and the MOAA/S score was 5, an additional dose of 0.5–1 mg of remimazolam or midazolam was administered in each group. Neither the midazolam group nor the remimazolam group used analgesics.

#### After the start of endoscopy

An endoscopist with at least 5 years of experience performed all colonoscopies using PCF‐260Z (Olympus). Vital signs (blood pressure, heart rate, and respiratory rate) and SpO_2_ were measured at 5‐min intervals. MOAA/S score was also rechecked at 5‐min intervals after the start of the examination: if the MOAA/S score was ≤4, the examination was continued, and if it was 5, an additional dose of sedative was administered. If signs of arousal (e.g., MOAA/S score 5, body movements) were observed at intervals other than 5 min, an additional sedative dose was administered at intervals of at least 2 min from the start of the immediately preceding dose.

#### After completion of endoscopy

After colonoscopy, vital signs (blood pressure, heart rate, and respiratory rate), SpO_2_, and MOAA/S scores were assessed at the end of the endoscopy, 5 min later, and at 10‐min intervals after 10–60 min. The patient's ambulatory ability was assessed if his/her MOAA/S score was 5. Patients were deemed capable of ambulating if they could walk in a straight line for 5 m without wobbling. Nurses monitored the midazolam group at the bedside after the endoscopy was completed, measuring vital signs (blood pressure, heart rate, and respiratory rate) and SpO_2_ at 5–10‐min intervals. Patients from both groups were discharged if the SpO_2_ exceeded 92%, systolic blood pressure surpassed 90 mmHg, they were awake, and ambulation was possible.

#### Definition of adverse events

In this study, adverse events associated with sedatives were investigated from the time of drug administration until the patient left the endoscopy room. The severity of adverse events was determined based on CTCAE, and changes in vital signs requiring medical intervention of grade 2 or higher were treated as adverse events associated with sedatives. Medical interventions were performed in both the midazolam and remimazolam groups according to the protocol of the REM‐IICT JP01 study. If systolic blood pressure was ≤90 mmHg on two consecutive occasions, it was determined to be hypotension, and fluid loading or ephedrine was administered. If Sp0_2_ was <94%, it was defined as hypoxemia and appropriate verbal encouragement was given, and 2–5 L of oxygen was administered. If the respiratory rate fell below 10 breaths, it was determined to be respiratory depression and appropriate verbal encouragement was given, and if the respiratory rate fell below 5 breaths, airway management (head tilt‐chin lift maneuver) was performed. If breathing did not improve despite airway management, manual ventilation was performed using a bag valve mask. Flumazenil was administered intravenously if emergency antagonism of sedation from the trial drug was needed.

### Outcomes

The primary outcome was the time from the end of the colonoscopy until discharge. The secondary outcomes were the time from the end of the colonoscopy until awakening, a dose before the colonoscopy, an additional dose, the total dose, and adverse events.

### Statistical analysis

Continuous variables were presented as medians (interquartile ranges) and compared using the Mann–Whitney U test. Categorical variables were compared using the Fisher test or χ^2^ test. Because this was a retrospective study, and the eligibility criteria differed slightly between the two groups, propensity score matching was employed to eliminate confounding factors. The two groups were matched in a 1:1 ratio with propensity‐score matching adjusted for five covariates

Age, sex, BMI, American Society of Anesthesiologists (ASA) classification, and examination time, which are related to the efficacy and safety of the sedatives, were used as factors in the propensity score using logistic regression analysis. This model yielded a C statistic of 0.78, indicating a preferable ability for comparison between the two groups. The caliper width for propensity score matching was set at 0.20. Statistical significance was set at *p* <0.05. All statistical analyses were performed using JMP (version 13.0.0; SAS Institute).

## RESULTS

### Before propensity score matching

In both the remimazolam and midazolam groups, all patients achieved a MOAA/S score of ≤4 before colonoscopy, and sedation was successful.

Table 1 shows the baseline characteristics of both groups before propensity score matching.

**TABLE 1:** Before propensity score matching: Background factors.

	Midazolam group (*N* = 206)	Remimazolam group (*N* = 60)	*p*‐value
Age (years)	62.0 (45.0–72.0)	53 (43.0–63.5)	<0.01
Sex			
Male	65 (31.3%)	20 (50.0%)	
Female	143 (68.7%)	20 (50.0%)	0.06
Height (cm)	165.0 (158.9–170.0)	163.3 (157.9–170.0)	0.54
Weight (kg)	62.2 (54.9–73.5)	60.4 (49.8–66.6)	0.10
BMI	23.2 (20.9–25.9)	22.4 (20.6–24.3)	0.13
ASA class, *n* (%)			
I	43 (20.7%)	21 (52.5%)	<0.01
II	141 (67.8%)	19 (47.5%)	
III	24 (11.5%)	0 (0%)	
IV	0 (0%)	0 (0%)	
V	0 (0%)	0 (0%)	

Data are presented as median (interquartile range) or number (percentage).

Abbreviations: ASA, American Society of Anesthesiologists; BMI, body mass index.

Patients in the remimazolam group (group R) were significantly younger at 53.0 (43.0–63.5) years than those in the midazolam group (group M) at 62.0 (45.0–72.0) years (*p* < 0.01). There were no statistically significant differences in height, weight, or BMI between both groups.

The distribution of the ASA status was as follows: class I (*n* = 43, 20.7%), class II (*n* = 141, 67.8%), and class III (*n* = 24, 11.5%) in the midazolam group; and class I (*n* = 21, 52.5%), class II (*n* = 19, 47.5%), and class III (*n* = 0, 0%) in the remimazolam group.

Table 2 shows the comparison of the efficacy of sedation between the two groups. The time from the end of the colonoscopy until awakening was 27.0 (10.0–34.0) min in the midazolam group and 0 (0–0) min in the remimazolam group; moreover, time from the end of the colonoscopy until discharge was 40.0 (35.0–48.0) min in the midazolam group and 0 (0–5.0) min in the remimazolam group, both of which were significantly shorter in the remimazolam group (*p* <0.01).

**TABLE 2:** Before propensity score matching: Efficacy and safety of sedation.

	Midazolam group (*N* = 206)	Remimazolam group (*N* = 60)	*p*‐value
Pre‐colonoscopy dose (mg)	2.0 (2.0–2.5)	3.0 (3.0–4.0)	
Number of additional doses (times)	0 (0–0)	2 (1–3)	<0.01
Total dose (mg)	2.0 (2.0–3.0)	6.0 (5.0–7.0)	
Examination time (min)	15.0 (11.0–20.0)	12.0 (11.0–14.0)	<0.01
Time from the end of colonoscopy until awakening (min)	27.0 (10.0–34.0)	0 (0–0)	<0.01
Time from the end of colonoscopy until discharge (min)	40.0 (35.0–48.0)	0 (0–5.0)	<0.01
Adverse events, *n* (%)	0 (0%)	1 (1.7%)	0.23
Hypotension	0 (0%)	0 (0%)	1.0
Hypoxemia (SpO_2_ <94%)	0 (0%)	1 (1.7%)	0.23
Respiratory depression	0 (0%)	0 (0%)	1.0

Data are presented as median (interquartile range) or number (percentage).

The number of pre‐colonoscopy doses was 2.0 (2.0–2.5) and 3.0 (3.0–4.0) mg in the midazolam and remimazolam groups, respectively; the frequency of additional doses was 0 (0–0) in the midazolam group and 2 (1–3) in the remimazolam group. The total dose was 2.0 (2.0–3.0) mg in the midazolam group and 6.0 (5.0–7.0) mg in the remimazolam group. The examination time was 15.0 (11.0–20.0) min for the midazolam group and 12.0 (11.0–14.0) min for the remimazolam group. Although one patient in the remimazolam group required oxygen administration, no patients in either group required flumazenil administration or manual ventilation during colonoscopy.

### After propensity score matching

Table 3 shows the background factors of both groups after propensity score matching. Thirty‐seven patients in both groups were matched. There were no statistically significant differences in background factors such as age, sex, BMI, and ASA classification between the two groups.

**TABLE 3:** After propensity score matching: Background factors.

	Midazolam group (*N* = 37)	Remimazolam group (*N* = 37)	*p*‐value
Age (years)	56.0 (48.5–62.0)	53.0 (44.0–64.0)	0.66
Sex, *n* (%)			
Male	26 (70.3%)	18 (48.7%)	
Female	11 (29.7%)	19 (51.3%)	0.09
Height (cm)	166.3 (161.8–173.5)	162.0 (161.8–170.9)	0.10
Weight (kg)	61.9 (52.1–75.5)	59.2 (53.2–66.9)	0.30
BMI	22.6 (19.7–26.2)	22.4 (20.7–24.4)	0.89
ASA class, *n* (%)			
I	21 (56.8%)	18 (48.7%)	0.11
II	13 (35.1%)	19 (51.3%)	
III	3 (8.1%)	0 (0%)	
IV	0 (0%)	0 (0%)	
V	0 (0%)	0 (0%)	

Data are presented as median (interquartile range) or number (percentage).

Abbreviations: ASA, American Society of Anesthesiologists; BMI, body mass index.

Table 4 shows the results of sedation efficacy of the two groups after propensity score matching. The time from the end of the colonoscopy until awakening was 28.0 (13.0–37.0) min in the midazolam group and 0 (0–0) min in the remimazolam group; moreover, Time from the end of the colonoscopy until discharge was 40.0 (35.0–46.5) min in the midazolam group and 0 (0–5.0) min in the remimazolam group, both of which were significantly shorter in the remimazolam group (*p* < 0.01).

**TABLE 4:** After propensity score matching: Efficacy and safety of sedation.

	Midazolam group (*N* = 37)	Remimazolam group (*N* = 37)	*p*‐value
Pre‐colonoscopy dose (mg)	2.0 (2.0–3.0)	3.0 (3.0–4.0)	
Number of additional doses (times)	0 (0–0)	2 (1–3)	<0.01
Total dose (mg)	2.0 (2.0–3.5)	6.0 (5.0–7.0)	
Examination time (min)	12.0 (10.0–14.5)	12.0 (11.0–14.0)	0.75
Time from the end of the colonoscopy until awakening (min)	28.0 (13.0–37.0)	0 (0–0)	<0.01
Time from the end of the colonoscopy until discharge (min)	40.0 (35.0–46.5)	0 (0–5.0)	<0.01
Adverse events, *n* (%)	0 (0%)	1 (2.7%)	1.0
Hypotension	0 (0%)	0 (0%)	1.0
Hypoxemia (SpO_2_ <94%)	0 (0%)	1 (2.7%)	1.0
Respiratory depression	0 (0%)	0 (0%)	1.0

Data are presented as median (interquartile range) or number (percentage).

Pre‐colonoscopy doses were 2.0 (2.0–3.0) mg in the midazolam group and 3.0 (3.0–4.0) mg in the remimazolam group. The number of additional doses was 0 (0–0) in the midazolam group and 2 (1–3) in the remimazolam group. The total dose was 2.0 (2.0–3.5) mg in the midazolam group and 6.0 (5.0–7.0) mg in the remimazolam group. The examination time was 12.0 (10.0–14.5) min for the midazolam group and 12.0 (11.0–14.0) min for the remimazolam group.

## DISCUSSION

To the best of our knowledge, this study is the first to compare remimazolam and midazolam sedation for colonoscopy in Japanese patients. Results have shown that remimazolam yielded a significantly faster time to awakening and time to discharge compared to midazolam. Because this was a retrospective study, propensity score matching was performed for the midazolam and remimazolam groups, as both agents were used at the same period in the same facility but in patients with disparate backgrounds. Remimazolam was associated with a shorter recovery time than midazolam. Importantly, few adverse events were observed with remimazolam, indicating that they can be used safely to deliver sedation in patients undergoing colonoscopy.

Few studies have compared remimazolam and midazolam for sedation during colonoscopy.

A phase III study conducted in the United States that compared the efficacy and safety of remimazolam and midazolam for colonoscopy found that the time from completion of the examination to awakening was 7.35 min in the remimazolam group versus 15.84 min in the midazolam group. That study also reported that the time from the end of the examination to discharge was 49.78 min in the remimazolam group and 57.44 min in the midazolam group, with significantly shorter recovery times in the remimazolam group.^18^ Additionally, a study comparing midazolam and remimazolam in high‐risk patients with ASA class III/IV found that the time to awakening was 3.0 min in the remimazolam group and 7.0 min in the midazolam group.^9^ A post‐hoc integrated analysis combining three clinical trials reported that the time to awakening was significantly shorter in the remimazolam group (3 min) than in the midazolam group (8 min), similar to our results.^20^ Use of fentanyl as an analgesic in the previous study may have caused the longer time to discharge in the patients administered remimazolam compared to our study because fentanyl may have acted synergistically with remimazolam.

Remimazolam reportedly has a good safety profile and causes no increase in adverse events compared to midazolam.^18^, ^19^, ^20^ In this study, none of the patients in either group required administration of flumazenil, a benzodiazepine antagonist. Furthermore, remimazolam has also been reported to significantly reduce medical costs by shortening the recovery time compared to midazolam.^19^ Furthermore, shortening the recovery time reduces the workload of medical staff in the recovery room.

There is currently a shortage of anesthesiologists in Japan, and sedation is administered by endoscopists. Because the use of propofol is restricted to anesthesiologists, it is not freely available, and midazolam is used primarily to induce sedation. Remimazolam is superior to midazolam in terms of efficacy, safety, and medical cost and may become the mainstay of endoscopic sedation in the future.

This study had several limitations. First, this was a retrospective study, and the remimazolam group was derived from the sample of a clinical trial; moreover, its background factors differed from those of the midazolam group. The remimazolam group targeted relatively healthy patients who could be enrolled in investigator‐initiated clinical trials. Therefore, we employed propensity score matching to adjust for background factors. We found that the time to awakening and time to discharge from the examination room was shorter in the remimazolam group than in the midazolam group. A multicenter, randomized, prospective, comparative study should be conducted in the future to validate this finding. Second, our study population was small and limited to patients of Japanese ethnicity. The results of this study cannot be generalized to all ethnic groups, warranting further studies with a larger population and multiple ethnicities.

## CONCLUSION

Compared to midazolam, remimazolam was found to reduce the time to awakening and time to discharge after the colonoscopy without increasing the incidence of adverse events.

## CONFLICT OF INTEREST STATEMENT

Author Hisatomo Ikehara received honoraria for his lectures from FUJIFILM Corporation, Mitsubishi Tanabe Pharma Corporation, and Olympus Corporation.

## ETHICS STATEMENT

This study was approved by the hospital's institutional review board and conducted in accordance with the Declaration of Helsinki. Informed written consent was obtained from all participants before the start of endoscopy.
