# Correction to “Comparison of remimazolam and midazolam for sedation during colonoscopy in Japanese patients: A propensity score matching analysis”

**DOI:** 10.1002/deo2.70059

**Published:** 2025-01-20

Ogura K, Ichijima R, Ikehara H et al. Comparison of remimazolam and midazolam for sedation during colonoscopy in Japanese patients: A propensity score matching analysis. *DEN Open* 2024; **5**: e412.

In Tables 1 and 2, the *N* = 206 for the midazolam group and the *N* = 60 for the remimazolam group are incorrect. The correct numbers are *N* = 208 and *N* = 40. Accordingly, the adverse event rate for the remimazolam group in Table 2 is *n* = 1, 2.5%, and the correct *p*‐value is 0.16.

The correct Table 1 and Table 2 are as follow.

**TABLE 1:** Before propensity score matching: Background factors.

	Midazolam group (*N* = 208)	Remimazolam group (*N* = 40)	*p*‐value
Age (years)	62.0 (45.0–72.0)	53 (43.0–63.5)	<0.01
Sex			
Male	65 (31.3%)	20 (50.0%)	
Female	143 (68.7%)	20 (50.0%)	0.06
Height (cm)	165.0 (158.9–170.0)	163.3 (157.9–170.0)	0.54
Weight (kg)	62.2 (54.9–73.5)	60.4 (49.8–66.6)	0.10
BMI	23.2 (20.9–25.9)	22.4 (20.6–24.3)	0.13
ASA class, *n* (%)			
I	43 (20.7%)	21 (52.5%)	<0.01
II	141 (67.8%)	19 (47.5%)	
III	24 (11.5%)	0 (0%)	
IV	0 (0%)	0 (0%)	
V	0 (0%)	0 (0%)	

*Note*: Data are presented as median (interquartile range) or number (percentage).

Abbreviations: ASA, American Society of Anesthesiologists; BMI, body mass index.

**TABLE 2:** Before propensity score matching: Efficacy and safety of sedation.

	Midazolam group (*N* = 208)	Remimazolam group (*N* = 40)	*p*‐value
Pre‐colonoscopy dose (mg)	2.0 (2.0–2.5)	3.0 (3.0–4.0)	
Number of additional doses (times)	0 (0–0)	2 (1–3)	<0.01
Total dose (mg)	2.0 (2.0–3.0)	6.0 (5.0–7.0)	
Examination time (min)	15.0 (11.0–20.0)	12.0 (11.0–14.0)	<0.01
Time from the end of colonoscopy until awakening (min)	27.0 (10.0–34.0)	0 (0–0)	<0.01
Time from the end of colonoscopy until discharge (min)	40.0 (35.0–48.0)	0 (0–5.0)	<0.01
Adverse events, *n* (%)	0 (0%)	1 (2.5%)	0.16
Hypotension	0 (0%)	0 (0%)	1.0
Hypoxemia (SpO_2_ < 94%)	0 (0%)	1 (2.5%)	0.16
Respiratory depression	0 (0%)	0 (0%)	1.0

*Note*: Data are presented as median (interquartile range) or number (percentage).

We apologize for this error.

